# A Paper Read before the Chicago Medical Society

**Published:** 1867-11

**Authors:** Hiram Wanzer

**Affiliations:** Chicago, Ill.


### A PAPER READ BEFORE THE CHICAGO MEDICAL SOCIETY.

By Hiram Wanzer, M.D., Chicago, Ill.

*Mr. President and Gentlemen:*—The subject of this paper is the therapeutical advantage of *position* in the treatment of shock from concussion of the brain, and I wish to report a case successfully treated by it.

During the past six years' residence in the West Division of our City, I have frequently been called to cases of traumatic injuries of the head. A few have succumbed to the shock without reaction, and, I apprehend, could we have had a clearer knowledge of our therapeutic agents, death might frequently have been postponed, and the fatal issue prevented. Since the suggestion made here by Dr. Orren Smith, to give the patient the advantage of *position*, I have tried it in three cases, two of which were of most desperate character, with the satisfaction of witnessing the recovery of them all. I can bear testimony to its priceless value. I believe the practice of surgery ought, and soon will be, revolutionized in the treatment of shock from concussion of the brain. Authors have not only failed to give, to my satisfaction, a clear and comprehensive idea of the pathological condition in concussion, occurring in the ordinary accidents of civil life, and, to me, their treatment is equally meagre and unsatifactory. I have been led to regard all injuries about the brain with significance. It was justly said, by Robert Liston, that no injury about the head was too trivial to be despised. It matters not whether the physical leison is well defined. Such as fracture of the cranium, with or without compression of the brain, by bone or blood-clot. Where there is much shock, or comatose symptoms, the organic contractility of the heart is enfeebled, if not nearly suspended. We find in this state of things, *position*, not only always indicated, but frequently the sheet-anchor upon which our patient may cling to the bark of life. This was exemplified among soldiers who fell in battle in our army. Those falling upon hill sides, with the head and shoulders dependent, usually recovered; those who fell with them elevated, frequently died.

The most prominent symptoms I have witnessed, in severe concussion of the brain are, general pallor, coldness, and anæsthesia of the surface of the body; insensibility of the organs of special sensation; the glassy eye usually does not respond to light; the ear may be deaf to sound; the organs of taste, touch, speech, and deglutition, obtunded, or nearly suspended, in proportion to the lesion to the brain and nerve centres. The heart's action is invariably feeble. The pulse, if it has not already forsaken the wrist, indicates the diminished contractile energy of the heart to carry the blood to the brain and extremities, and, without speedy help, our patient is soon beyond the surgeon's skill.

Having repeatedly witnessed the insufficient action of remedies in the treatment of shock, my practice has not only become radically changed, but, I believe, based upon more physiological and rational principles. Our most certain means are always at hand, and if early resorted to, will generally extricate our patient from impending death. The *rationale* of treatment is precisely the same that we would adopt in the parturient woman, suffering from uterine hemorrhage: first, to restore as speedily as possible, to the brain, its lost stimulus, by *position*, placing our patient at an angle of forty-five degrees, with the hips elevated and the head dependent. By this method, the emptied vessels of the brain are refilled, the heart soon responds to the reflex nervous influence; its motion and rhythm reëstablished; the forsaken pulse returns to the wrist with force and volume. Thus does the brain minister to organic action when it has its accustomed supply.

There are other collateral, but auxilliary therapeutic agents, that now come to his relief, some of which I will mention. In cases of profound shock to the nervous system, the patient is usually incapacitated from swallowing, and the measures I have adopted are to give remedies by enema, in liquid form, first placing our patient in *position*, hot epithems are applied to the extremities, and from one-half to one ounce of brandy, with five grains gum camphor, diluted with a small quantity of water, are thrown into the rectum, repeated, if necessary, at intervals of half an hour, according to the exigency of the case. As soon as the heart's action become more vigorous, and the pulse returns at the wrist, with moderate force and fulness, with the capiliary circulation established, the head and shoulders are slightly elevated. He is continued in this *position* until some degree of consciousness is restored, when he is placed in the recumbent posture, resuming the former *position*, upon the first approach to fatal syncope, indicated by the flagging pulse and diminution of the heart's action. This I found necessary to resort to in one case, to the salvation of my patient. A valuable adjuvant in the treatment, are jets of cold water, thrown with the fingers into the face, assisted by smelling salts, which stimulate the trifacial nerve.

I have tested the value of the above methods of treatment in three cases, as before stated, who would have perished under previous methods I have seen adopted.

I believe, in the treatment of shock, narcotics are seldom if ever indicated. They not only diminish the vital powers, obtund and paralyze nerve sensibility, and enfeeble the energies of the *tripod of life*, but they deprive our patient of *pain*, a natural stimulant and excitor to organic action, in the beginning reaction. The true indications of treatment are, first, to restore the wasted energies of the brain and nerves centre, from whence organic action emanates by *position*. Second, diffusible stimulants, medicines that exalt the vital actions of the economy, combined or alternated with beef essence.

The case of traumatic injury of the head, I wish to report this evening, was Cornelius Daily, native of Ireland, aged 22. I was summoned to him May 17th, last, at 8 A.M. Before my arrival at the scene of excitement, I was met by a messenger, who stated that the man was dead, but hastening to the spot, I found the patient lying upon the ground. He had been struck upon the left side of the head by the fall of a timber weighing some 200 lbs. The wound was contused, and extended from the zygoma to the mastoid process of the temporal bone. The lobule of the ear was also divided. There was a large quantity of dark coagulated blood upon the ground where he was lying, which had issued from his mouth, nose, and ears. Upon examination, I found the surface of the body cold. The eye was glassy, and did not respond to light. There was no pulse at the wrist. With the exception of an indistinct tremor of the heart, no defined sound being audible, life was apparently extinct. An assistant now seized the hips of the patient, elevating them considerably, leaving the head down. In about ten minutes, the heart's action became more perceptible, and distinct. Two planks were now arranged at an angle of forty-five degrees, and the patient placed upon them with the head and shoulders dependent. As soon as he was placed in *position*, nearly a pint of dark coagulated blood issued from the mouth, nose, and ears. In about fifteen minutes, the pulse returned to the wrist. The contractile energy of the heart was more manifest. The gentle glow of the surface indicated the beginning return of the capillary circulation. Cold water was now dashed upon the face, alternated by jets with the fingers. We found this of inestimable value in the treatment. Believing that he would fully react, the divided ear was coaptated by several interrupted sutures. He was continued in *position*, about thirty minutes longer, when the heart's action and pulse became quite vigorous, with a more marked capillary circulation. He was now removed to the warehouse of Messrs. S. S. Williamson & Co., and placed in nearly the same *position*, the head and shoulders being slightly elevated. Upon his arrival there he vomited a quantity of dark grumous blood, which somewhat increased his prostration. Hot epithems were now applied to the extremeties. The body was enveloped in hot flannel blankets; an enema of half an ounce of spirits vini. gallicis, with five grains gum camphor, diluted with a small quantity of water, was thrown into the rectum. This was repeated every hour during the forenoon. In the afternoon, consciousness, sight, speech, and deglutition partially returned; but reaction had been somewhat retarded by *paroxysms* of vomiting, which consisted of the fluids he had drank, mingled with blood. This was relieved by acid gallici, giving five grains after each effort at vomiting. At 7 P.M., a more comfortable reaction was established. He had slept considerably during the afternoon. His breathing was noisy and stertorous; had two fecal evacuations; the surface of the body was about the normal temperature. As there was now but little difficulty in deglutition, the enemas were omitted, and he was ordered for the night, every three hours, two grains gum camphor, alternated with beef essence.

The 18th, at 7 A.M. The patient had enjoyed a few hours of refreshing sleep; there had been less stertor; the mind was more clear. He had been annoyed considerably by thirst, which I ascribed to the excessive loss of blood. He had several fecal discharges during the night. There was no change in the treatment during his sojourn there, but simply to continue *position*, the external warmth, and the beef essence. He was removed to the City Hospital in an ambulance at 3 P.M., and to the very kind and skilful treatment of the Surgical Staff of that institution, he owes much to, and for, his recovery. I will now read the Hospital Report from the time of his admission there:—

Cornelius Daily, *æt* 22 years; a native of Ireland; stature, a little less than medium. While engaged as a laborer, assisting in the removal of some machinery off the cars, was felled to the ground by a blow from a heavy piece of timber, on the left side of the head, immediately over the ear.

There soon followed copious hemorrhage from the nares and ears, when he afterwards lay comatose, completely insensible, for several hours.

On May 18th, the first succeeding day of his injury, he was admitted into Cook County Hospital presenting the following symptoms:—

Although he answered interrogatories with seeming correctness, he still exhibited a mental lethargy often seen in patients semi-comatose. He was unable to maintain the erect posture, either in sitting or standing, and when placed in bed, kept, without moving, as perfect dorsal decubitus, as if wholly paralyzed, but when requested to move, there was no apparent difficulty or disparity in the natural mobility of any extremity.

The face gives evidence of recent severe contusion, extending from the left eye to the ear of the same side. The nasal and aural canals were filled with clotted blood; the pinna of the left ear was lacerated, and had been dressed with sutures. The right pupil was normal, the left, moderately dilated, sphincters enjoying their usual tonicity; bowels constipated.

He slept during the first forty-eight hours almost continually; ate but sparingly of anything. About the fourth day the external recti muscles were seen to be paralyzed on both sides. The left cornea perfectly anæsthetized, and the sensation of the right markedly obtuse. The conjunctiva of both sides exhibited deficient sensation; the left exceeding in degree. With the instillation of a drop of a four grain solution of atropia sulph., ready dilation of both pupils followed.

Both corneæ had now begun to ulcerate. The ulcer on the right progressed but slightly, but the left proceeded to perforation and evacuation of the anterior chamber entirely, as was evinced by the perfect approximation of the iris and inner wall of the cornea.

The treatment consisted in keeping both pupils persistently dilated, simultaneously sealing the left eye-lids with isinglass plaster, as the lower palpebræ drooped, so as to leave the eye continually open. The paralysis involved all the muscles of the left side of the face, with the loss of the sense of taste over the left half of the tongue; also, convergent strabismus of both eyes. The corneal ulcers healed steadily under the treatment employed, and as they cicatrized, the anæsthesia diminished. The perforation of the left cornea soon closed, and a new secretion of aqueous humor restored its original convexity. He was transferred to the eye and ear wards, June 19th, 1867, having, at the time, a purulent discharge from the left ear, with inflammation of the conjunctiva of the corresponding eye, kept up by the lost tone in the orbicularis, which exposes the unprotected mucous surface to the atmosphere. Under appropriate treatment and cleanliness, the ear has ceased to suppurate; the left eye is less inflamed, the right being entirely well. Both eyes are thoroughly strabismic; but very recently, there is a manifest return of motion to the external rectus of the left. The facial paralysis of this side remains still the same, while the arm and leg of the opposite half of the body exhibit deficient nerve power.

